# VEGF levels in plasma in relation to metabolic control, inflammation, and microvascular complications in type-2 diabetes: A cohort study

**DOI:** 10.1097/MD.0000000000010415

**Published:** 2018-04-13

**Authors:** Qin Zhang, Wen Fang, Li Ma, Zhao-Di Wang, Yun-Mei Yang, Yuan-Qiang Lu

**Affiliations:** Department of Emergency and Geriatrics Medicine, The First Affiliated Hospital, School of Medicine, Zhejiang University, Hangzhou, ZheJiang, China.

**Keywords:** diabetic microvascular complication, T lymphocyte, type-2 diabetes mellitus, vascular endothelial growth factor

## Abstract

The vascular endothelial growth factor (VEGF) level in human circulation may reflect the severity of endothelial dysfunction in patients with diabetes mellitus, which leads to diabetic microvascular complications.

We determined plasma VEGF levels as well as metabolic control and inflammatory factors in 26 healthy subjects and 52 type-2 diabetes mellitus (T2DM) patients with or without diabetic microvascular complications. Pearson correlation coefficient was used to evaluate the associations among those indices.

The results showed that VEGF levels in plasma were positively correlated with fasting blood glucose level, glycosylated hemoglobin (HbA1c) level, type 1 helper T cell (Th1) percentage, and Th1/Th2 ratio, while they were negatively correlated with regulatory T cell percentage. Multiple linear regression analysis showed that HbA1c and Th1/Th2 ratio were the independent predictors of VEGF levels in T2DM patients.

Thus, in T2DM patients with poor glycemic control as well as an elevated Th1/Th2 cell ratio, more VEGF might be released.

Individuals with type-2 diabetes mellitus (T2DM) are often at high risk for microvascular complications, including diabetic retinopathy (DR), diabetic nephropathy (DN), and diabetic peripheral neuropathy (DPN) due to diabetic microvascular dysfunction.^[1,2]^ Angiogenesis, as an essential biological process, involves the progression of diabetic microvascular complications. Meanwhile, vascular endothelial growth factor (VEGF) is the most potent proangiogenic growth factor that increases vascular permeability in vivo and activates endothelial cells in vitro.^[3]^ Taken together, VEGF may play an important role in diabetic endothelial dysfunction, which leads to diabetic microvascular complications. Previous studies have found that VEGF is involved in the pathogenesis of diabetic complications.^[4–7]^ Plasma VEGF levels were reported to be higher in diabetic patients than in healthy control individuals, and a correlation of plasma VEGF levels with proliferative DR and DN has also been noticed.^[8,9]^ The synthesis and secretion of VEGF are affected by several factors, including gender, hypoxia, hyperglycemia, smoking, blood lipids, inflammatory reaction, and activated stress axes.^[10]^ However, there are few research studies that illustrate the detailed mechanism of the association between VEGF and diabetic microangiopathy, particularly in T2DM.^[11]^ To better understand it, T2DM patients with or without diabetic microvascular complications and healthy volunteers were selected, and their VEGF plasma levels as well as other clinical parameters were assessed in this study. We investigated the relationship between plasma VEGF levels and parameters of metabolic control, inflammation, and the presence of diabetic microvascular complications.

## 1 Methods

### 1.1 Subjects

The study included 26 healthy volunteers as a control group (category 1, n = 26) and 52 patients diagnosed with T2DM under the care of the First Affiliated Hospital, School of Medicine, Zhejiang University, China between October 2015 and December 2016. The T2DM patients were divided into 2 categories: T2DM without microvascular complications (category 2, n = 26) and T2DM with microvascular complications (category 3, n = 26). Twenty-six individuals were selected in category 3, including 1 case of DR, 7 cases of DN, 11 cases of DPN, 3 cases of DR combined with DPN, 2 cases of DR combined with DN, and 2 cases of DPN combined with DN. To minimize the statistical error caused by small samples, we put all T2DM patients with various diabetes microvascular complications into category 3.

To qualify for the study, patients had to satisfy the following criteria: a level of glycosylated hemoglobin (HbA1c) of ≥6.5% and fasting blood glucose (FBG) ≥7.0 mmol/L, or with a glucose tolerance test, 2 h after the oral dose, a plasma glucose level ≥ 11.1 mmol/L. Patients meeting any of the above criteria can be categorized as T2DM. The control category selected healthy participants without any type of diabetes, as well as without hypertension, hyperlipidemia, and other metabolic syndromes. DR was diagnosed according to the Clinical Guidelines of Diabetic Retinopathy in China (2014).^[12]^ DN was diagnosed when albuminuria > 300 mg/24 h, or glomerular filtration rate < 60 mL/min lasting for 3 months. DPN was diagnosed with the following symptoms: abnormal temperature sense, foot sense loss detected by nylon yarn, decreased vibration perception, disappeared ankle reflexes, and nerve conduction tests showing reduced functioning of the peripheral nerves. Patients matched 2 or more of the above clinical peripheral neuropathy and electrophysiological symptoms can be defined as DPN. T2DM patients who had serious medical comorbidities, such as macroangiopathy, acute inflammation, autoimmune diseases, endocrine diseases, malignancies, hematological diseases, unstable angina or myocardial infarction, end-stage cardiac insufficiency, cerebral infarction, and pulmonary or hepatic diseases, were excluded from this study.

This study was approved by the Medical Ethical Committee of First Affiliated Hospital, School of Medicine, Zhejiang University, and all participants gave written informed consent. The methods in this study were performed in accordance with the relevant guidelines and regulations.

### 1.2 Measurements of plasma VEGF, metabolic and inflammatory parameters

From each patient or healthy volunteer, the 10 mL of venous blood was collected from an elbow vein between 6:30 and 7:30 am in a fasting state. Blood samples were kept in 3 types of tubes. Six milliliters of blood was collected in a tube containing ethylenediaminetetraacetic acid to determine the plasma VEGF level and lymphocyte cell counts in peripheral blood. The blood sample was centrifuged for 10 min at 400g to obtain plasma. The plasma VEGF concentration was determined using human VEGF-A platinum enzyme-linked immunosorbent assay kit (eBioscience, Vienna, Austria). The remaining peripheral blood mononuclear cells were collected after sequentially adding RBC Lysis Buffer and Ficoll-Paque PLUS (Sigma-Aldrich, St Louis, MO) to remove the red blood cells and granulocytes for further lymphocyte cell analysis. A flow cytometer (FC500, FACSCalibur; Beckman-Coulter, Brea, CA) was used to detect regulatory T cells (Treg: CD4+CD25+FoxP3+), type 1 helper T cells (Th1: CD4+T-bet+), and type 2 helper T cells (Th2: CD4+GATA3+), and the ratio of Th1/Th2 cells was calculated. Th1 and Th2 antibodies used for flow cytometry were purchased from BD Biosciences (Franklin Lakes, NJ); Tregs antibodies were purchased from eBioscience, Inc. (San Diego, CA). In addition, 2 mL of blood was drawn into a tube containing sodium fluoride to determine the FBG and HbA1c. Another 2 mL of blood was collected in a serum tube, and the serum C-reactive protein (CRP) and homocysteine (Hcy) levels were then analyzed.

Clinically, FBG and HbA1c are the most widely used parameters for glycemic control. Hcy is the marker of amino acid metabolism disorder and has been demonstrated to be an independent risk factor for cardiovascular and cerebrovascular diseases.^[13,14]^ The CRP, Tregs, and Th1/Th2 ratios are the common clinical indicators for immune and inflammatory responses.

### 1.3 Statistical analysis

All statistical analyses were performed using SPSS version 18.0 software (SPSS Inc., Chicago, IL). The categorical variables are expressed as numbers or percentages, and the continuous variables that were close to a normal distribution are presented as the mean ± standard deviation. The chi-squared test was applied for comparisons of categorical variables. For continuous variables, the 1-way analysis of variance with the least significant difference *t* test was applied for comparisons differences among the 3 categories. Pearson correlation coefficients were calculated between the VEGF level and clinical indicators of metabolic control, and markers of inflammation. Forward multiple linear regression analysis was performed with VEGF levels as the dependent variable and those determinants that correlated in the univariate analysis with *P* < .05 as independent variables. For multiple comparisons, *P* < .05 was considered significant.

## 2 Results

### 2.1 General clinical data

Data were obtained from 78 participants (41 males and 37 females). The demographic and clinical parameters of 3 categories were recorded: gender, age, diabetes duration, body mass index (BMI), waist-to-hip ratio (WHR), comorbidity, and smoking habits (Table 1). No differences in sex ratio, age, or rate of smokers were found among the 3 categories (all *P* > .05). The diabetes duration in category 3 was longer than that in category 2 (*P* < .05). The WHRs were higher in the T2DM categories, but no difference was found between categories 2 and 3 (*P* > .05). BMI was elevated significantly in category 3 compared with category 1 (*P* < .05). The percentages of hypertensive patients among the 3 categories were significantly different (*P* < .001), but no difference was found between categories 2 and 3 (*P* > .05).

**Table 1:**
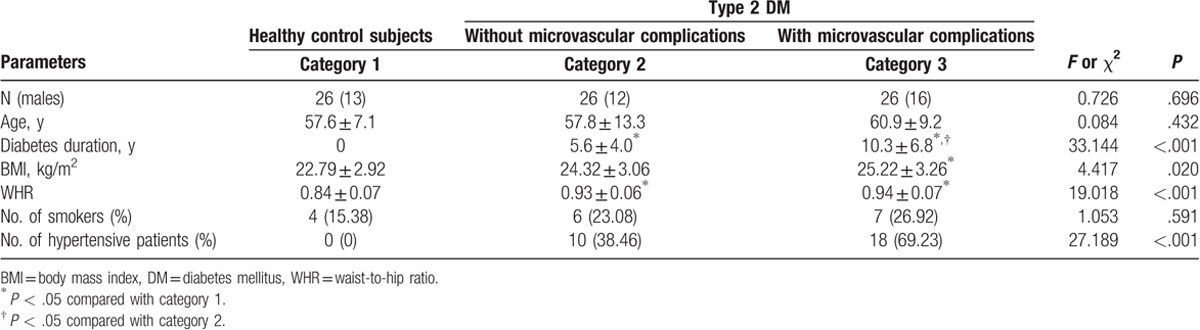
Demographic and clinical parameters according to category.

### 2.2 Metabolic and inflammatory parameters

The metabolic and inflammatory parameters of the 3 categories are summarized in Table 2. The indicators of glycemic control (FBG and HbA1c) in the T2DM categories were significantly higher than those in category 1 (all *P* < .05), but no difference was found between the 2 T2DM categories (all *P* > .05). In addition, the Hcy concentration was elevated significantly in category 3 compared with that in the other 2 categories (both *P* < .05). As markers of inflammation, Th1 percentages and Th1/Th2 ratio were significantly higher, as well as CD4+CD25+FoxP3+Treg percentages being lower in the 2 T2DM categories than in category 1 (all *P* < .05). In particular, the Th1 percentages and Th1/Th2 ratio in category 3 were even higher than in category 2 (all *P* < .05). There were no marked differences in Th2 percentages among all categories (*P* > .05). The CRP and plasma VEGF levels in the 2 T2DM categories were obviously higher than those in category 1; meanwhile, CRP and VEGF levels in category 3 were also higher than those in category 2 (all *P* < .05).

**Table 2:**
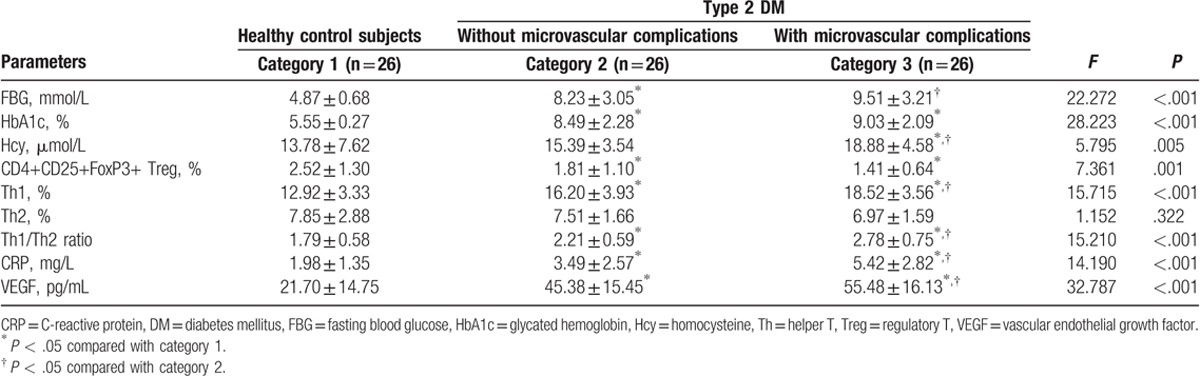
Metabolic and inflammatory parameters according to category.

### 2.3 Relationships between VEGF level and its putative determinants

**Figure 1:**
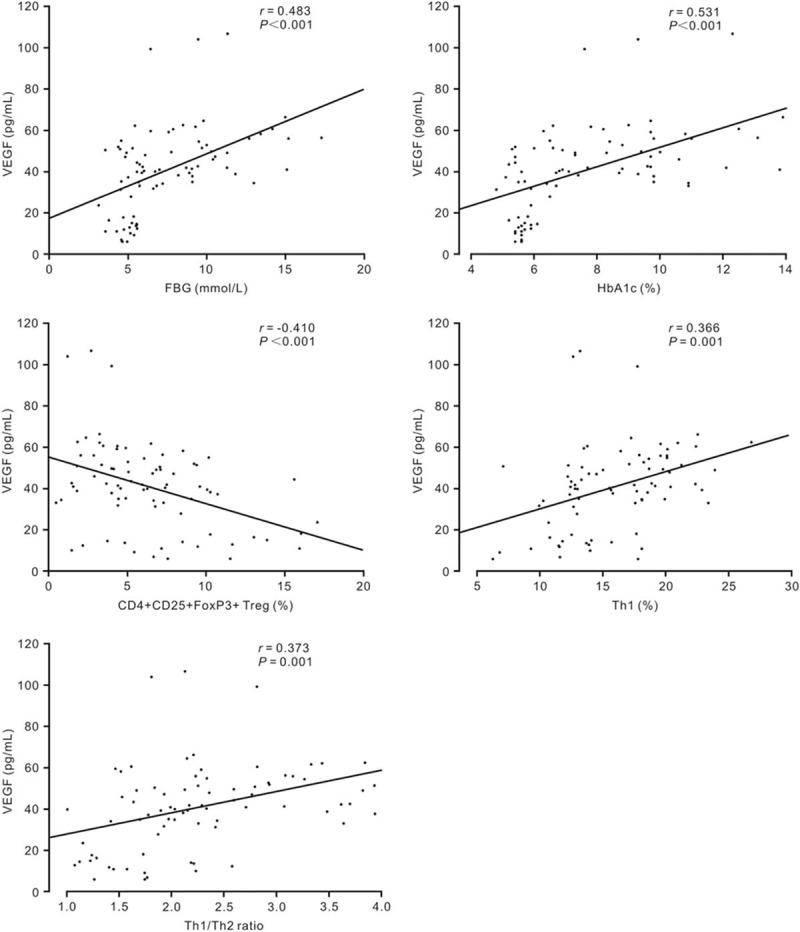
Relationship between VEGF levels in plasma and its putative determinants in healthy subjects and T2DM patients. FBG = fasting blood glucose, HbA1c = glycated hemoglobin, T2DM = type-2 diabetes mellitus, Th = helper T, Treg = regulatory T, VEGF = vascular endothelial growth factor.

Pearson correlation coefficients were calculated to analyze the associations between plasma VEGF level and clinical indicators of metabolic control or inflammation in healthy subjects and T2DM patients. Figure 1 demonstrated that VEGF levels were correlated positively with FBG level (*r* = 0.483, *P* < .001), HbA1c level (*r* = 0.531, *P* < .001), Th1 percentage (*r* = 0.366, *P* = .001), and Th1/Th2 ratio (*r* = 0.373, *P* = .001) in the T2DM patients and controls. By contrast, a negative correlation between the VEGF level and CD4+CD25+FoxP3+Treg percentage was also found. Multiple linear regression analysis indicated that only the HbA1c level and Th1/Th2 ratio were independent predictors of VEGF levels in T2DM. The regression equation of VEGF levels was VEGF = −5.275 + 4.099 × HbA1c + 6.471 × Th1/Th2 ratio (*r*^2^ = 0.332; *P* < .001). The residual sum of the squares indicated that 33.2% of variation in VEGF levels was explained by the HbA1c level and Th1/Th2 ratio.

## 3 Discussion

In this study of people with and without T2DM, we found that VEGF levels in plasma were positively correlated with glycemic control indicators (FBG and HbA1c), and inflammatory parameters (Th1, Th1/Th2 ratio), while they were negatively correlated with Treg percentage; further multiple linear regression analysis revealed that HbA1c and Th1/Th2 ratio were the independent predictors of VEGF levels in plasma of T2DM patients. This indicates a close association among hyperglycemia, inflammation, and VEGF in T2DM patients.

The number of diabetes cases worldwide is approximately 366 million according to the estimation by the International Diabetes Federation, of which T2DM constitutes approximately 90% to 95%.^[15]^ Up to 80% of mortality with diabetes is directly associated with vascular diseases affecting micro- or macrocirculation.^[16]^ Angiogenesis, about the formation and differentiation of blood vessels, is an essential biological process existing in embryogenesis and in the development of major diseases, such as cancer, inflammation, and diabetes.^[17]^ The fundamental regulator most widely known to be involved in angiogenesis is VEGF. VEGF is also associated with tumor progression and poor outcomes in various human cancers.^[18,19]^ The activated platelets and leukocytes are the main sources of VEGF in blood.^[20]^ In cultured endothelial cells, VEGF has been proven to be induced by the elevated levels of glucose and advanced glycation end products.^[11]^ It is interesting that the correlations of VEGF with blood glucose concentrations and immune levels are both found in our cohort study, suggesting an interaction may exist among hyperglycemia, inflammation, and VEGF.

Chronic hyperglycemia has been reported to stimulate the synthesis and secretion of VEGF-A. It triggers a chain reaction that contributes to VEGF-A accumulation and then leads to DM microvascular complications.^[21]^ The major physiological stimulus for VEGF production is cellular hypoxia. Hyperglycemia can act as toxin to the endothelium through increasing oxidative stress. The high concentration of blood glucose increases the production of vasoconstrictor substances, particularly endothelin-1.^[22]^ Hyperglycemia-induced pathological mechanism affects the expression of VEGF and its receptors VEGFR1 and VEGFR2. The elevated circulating VEGF-A levels are already found in adult T1DM patients with DN, and in T1DM prepubertal and pubertal children.^[8,23]^ Similarly, urinary VEGF-A was elevated in T2DM patients and the diabetic mice model holding no correlation with their albuminuria.^[24,25]^*VEGF-A* polymorphisms are associated with DR and DN as well.^[21]^ The characteristic parameter to evaluate glucose control in the blood is the level of HbA1c. Mahdy et al^[7]^ measured the serum VEGF level in T2DM patients before glycemic control and at 4 months follow-up and observed a significant decrease in the serum level of VEGF in patients with glycemic control.^[7]^ These previous research studies are in line with the results from our study, which show that a significant correlation exists between the concentrations of VEGF and glycemic control.

Hcy has been reported to inhibit endothelial cell proliferation and induce endothelial dysfunction as well as endothelial cell apoptosis.^[26–28]^ The serum Hcy concentration of T2DM with microvascular complications group was elevated significantly, compared with the 2 other groups in our research. These results suggest that there may be more serious endothelial damage and metabolic disorders in the T2DM patients with microvascular complications.

In our study, CRP, Th1 percentage, and Th1/Th2 ratio in the T2DM patients with microvascular complications were significantly higher than those in the control and T2DM without microvascular complications. This indicated that persistent inflammatory activity was involved in the progression of microvascular complications in diabetes. Inflammatory reaction can lead to an increase in vascular permeability, endothelial cell apoptosis, and chronic inflammation. Actually, CRP as common clinical indicator of inflammatory status could upregulate the VEGF-A expression by activating hypoxia inducible factor-1alpha in adipose-derived stem cells.^[29,30]^ Furthermore, disruption in immune homeostasis with a shift toward a Th2-dominant or chronic inflammatory state by tumor-derived VEGF has been reported previously.^[31]^ By contrast, we noticed that the Th1/Th2 immune imbalance with a shift to a Th1-dominant was associated with plasma VEGF accumulation in T2DM. The Th1/Th2 ratio has switched directions in diabetes possibly due to the diverse immune-related cytokines activated in these diseases and then triggered the proliferation of different helpers T cells. Treg is an indicator of immune response, which has the potent immunosuppressive function to maintain immune homeostasis.^[32,33]^ VEGF is proved to be a promoter of Treg activation in antitumor immunity. Conversely, a negative correlation between the plasma VEGF level and Treg percentage in T2DM was noticed in our study, although further analysis demonstrated that the Treg concentration was not an independent predictor of VEGF levels in T2DM. The above results showed that the inflammation levels of T2DM patients were higher than those of the healthy control group. Particularly in T2DM patients with microvascular complications, there may exist a more serious immune dysfunction.

In next multivariate analysis, only HbA1c and Th1/Th2 ratios were found to be independent determinants of the VEGF plasma level. The independent correlation between VEGF and HbA1c has been reported only in T1DM.^[11]^ Combined with our data, this observation suggests that in both T1DM and T2DM, poor glycemic control possibly leads to more VEGF released. We can also speculate that the possibility of higher release of VEGF in patients with poor glycemic control and persistent inflammation activity could be explored as a potential contributor to endothelial dysfunction in diabetic patients.

The limitations of this study are that the population of T2DM with microvascular complications patients is not large enough to be subdivided based on different types of complications. A large population can minimize statistical variance. Next step, we will verify if the elevated VEGF levels could be reversed after controlling the diabetic states of T2DM patients.

In conclusion, our study showed a close association among hyperglycemia, inflammation, and VEGF, which link with microvascular diseases in T2DM patients. The elevated circulating levels of Th1 and VEGF may contribute to the pathogenesis of T2DM microangiopathy.

## Author contributions

**Conceptualization:** Yuan-Qiang Lu.

**Formal analysis:** Qin Zhang, Li Ma.

**Funding acquisition:** Yun-Mei Yang, Yuan-Qiang Lu.

**Investigation:** Qin Zhang, Zhao-Di Wang, Yun-Mei Yang.

**Methodology:** Qin Zhang, Zhao-Di Wang, Yun-Mei Yang.

**Project administration:** Yuan-Qiang Lu.

**Software:** Li Ma.

**Supervision:** Yuan-Qiang Lu.

**Writing – original draft:** Wen Fang, Yuan-Qiang Lu.

**Writing – review & editing:** Qin Zhang, Wen Fang, Yuan-Qiang Lu.
